# Exploring the Potential Benefits of Natural Calcium-Rich Mineral Waters for Health and Wellness: A Systematic Review

**DOI:** 10.3390/nu15143126

**Published:** 2023-07-13

**Authors:** Manuela Simona Pop, Dragoș Cornel Cheregi, Gelu Onose, Constantin Munteanu, Cristina Popescu, Mariana Rotariu, Marius-Alexandru Turnea, Gabriela Dogaru, Elena Valentina Ionescu, Doinița Oprea, Mădălina Gabriela Iliescu, Mihaela Minea, Liliana Elena Stanciu, Sînziana Călina Silișteanu, Carmen Oprea

**Affiliations:** 1 Faculty of Medicine and Pharmacy, University of Oradea, 410073 Oradea, Romania; drpsimona@gmail.com (M.S.P.);; 2 Teaching Emergency Hospital “Bagdasar-Arseni” (TEHBA), 041915 Bucharest, Romania; cristina_popescu_recuperare@yahoo.com; 3 Faculty of Medicine, University of Medicine and Pharmacy “Carol Davila” (UMPCD), 020022 Bucharest, Romania; 4 Faculty of Medical Bioengineering, University of Medicine and Pharmacy “Grigore T. Popa” Iași, 700454 Iași, Romania; mariana.rotariu@umfiasi.ro (M.R.); marius.turnea@umfiasi.ro (M.-A.T.); 5 Faculty of Medicine, “Iuliu Hatieganu” University of Medicine and Pharmacy, 400012 Cluj-Napoca, Romania; dogarugabrielaumf@gmail.com; 6 Clinical Rehabilitation Hospital, 400437 Cluj-Napoca, Romania; 7 Faculty of Medicine, Ovidius University of Constanta, 900527 Constanta, Romania or elena_valentina_ionescu@yahoo.com (E.V.I.); doi_opr@yahoo.com (D.O.); iliescumadalina@gmail.com (M.G.I.); lilianastanciu77@yahoo.com (L.E.S.); carmen_oprea_cta@yahoo.com (C.O.); 8 Balneal and Rehabilitation Sanatorium of Techirghiol, 34-40 Dr. Victor Climescu Street, 906100 Techirghiol, Romania; mihaela_minea_2005@yahoo.com; 9 Faculty of Medicine and Biological Sciences, “Stefan cel Mare” University of Suceava, 720229 Suceava, Romania; sinziana.silisteanu@usm.ro

**Keywords:** natural calcium-rich mineral waters, calcium intake, bioavailability, dietary calcium, calcium in drinking water

## Abstract

This systematic review investigates the potential health and wellness benefits of natural calcium-rich mineral waters. It emphasizes the importance of dietary calcium sourced from natural mineral waters in promoting bone health, maintaining cardiovascular function, aiding in weight management, and enhancing overall well-being. The review process involved the comprehensive analysis of peer-reviewed articles, clinical trials, and experimental studies published within the last decade. Findings reveal that consuming calcium-rich mineral water can contribute significantly to daily calcium intake, particularly for those with lactose intolerance or individuals adhering to plant-based diets. The unique bioavailability of calcium from such waters also appears to enhance absorption, thus potentially offering an advantage over other calcium sources. The potential benefits extend to the cardiovascular system, with some studies indicating a reduction in blood pressure and the prevalence of cardiovascular diseases. Emerging evidence suggests that calcium-rich mineral water might have a role in body weight management, though further research is needed. The review identifies several areas requiring additional research, such as the potential interaction between calcium-rich mineral water and other dietary components, the effects on populations with specific health conditions, and the long-term effects of consumption. In conclusion, natural calcium-rich mineral waters show promise as a readily accessible and bioavailable sources of dietary calcium, potentially beneficial for a broad range of individuals. However, further investigation is required to fully understand its range of health impacts and define optimal intake levels.

## 1. Introduction

Calcium, the most prevalent mineral in the human body, is critical in maintaining the structure and vitality of our physical systems [1,2]. Most of the body’s calcium—about 99%—is stored within the bones and teeth, offering rigidity and sustaining their architecture. Notably, it contributes to the hardness of the enamel, the outermost layer of the teeth, which is the hardest substance in the human body. Therefore, adequate calcium intake is essential in maintaining dental health and preventing conditions like dental caries [3].

Adequate calcium intake levels vary based on age, sex, and life stage. As stated by the National Institutes of Health (NIH), the recommended dietary allowances (RDAs) for calcium are as follows: infants 0–6 months: 200 milligrams per day; infants 7–12 months: 260 milligrams per day; children 1–3 years: 700 milligrams per day; children 4–8 years: 1000 milligrams per day; children and adolescents 9–18 years: 1300 milligrams per day; adults 19–50 years: 1000 milligrams per day; men 51–70 years: 1000 milligrams per day; women 51–70 years: 1200 milligrams per day; adults 71 years and older: 1200 milligrams per day; pregnant and breastfeeding women: 1000–1300 milligrams per day (https://ods.od.nih.gov/factsheets/Calcium-HealthProfessional/ accessed on 1 July 2023).

In the skeletal system, calcium is a significant component of bone tissue, providing both density and strength. The bones act as a storage system for calcium; they continually uptake and release the mineral as required by the body. This dynamic process allows the skeletal system to maintain its structure and support the body, allowing for the mobility and protection of vital organs [4,5]. Importantly, this function of calcium is crucial at every stage of life—it supports rapid bone growth during childhood and adolescence, maintains bone health in adulthood, and helps slow bone density loss that comes with aging [6,7]. Arguably, calcium’s most recognized function is its contribution to bone health. This role is particularly crucial during rapid growth, such as childhood and adolescence. Preserving bone density remains a critical task in adulthood, serving as a preventive measure against osteoporosis. This condition is identified by the degradation of bone tissue and a reduction in bone mass [8,9].

Calcium’s significance extends well beyond its structural role [10]. It is a pivotal player in various biochemical reactions and physiological processes. Calcium ions act as a signal in many cellular processes [11]. For instance, the process of muscle contraction relies heavily on calcium. In response to a nerve signal, calcium ions are released within muscle cells, triggering a series of events that lead to the contraction of the muscle fibers, initiating a chain of events that allow the muscle proteins to ‘slide’ by one another and subsequently contract the muscle. The muscle relaxes when the calcium is pumped back into calcium cellular storage compartments. This highlights the importance of calcium in maintaining normal muscle function and heart rhythm. Without calcium, our muscles, including our heart—fundamentally, a muscle—would not be able to contract and relax properly [12,13].

Furthermore, calcium is essential in blood clotting, which is vital for healing wounds. In response to a wound, platelets create a plug at the wound site, and a series of reactions, many of which require calcium, occur to form a fibrin clot and prevent excessive bleeding. Coagulation, or blood clotting, is a complex process involving a series of chemical reactions. Calcium is a crucial co-factor for several enzymes involved in the clotting process. It aids in the conversion of prothrombin into thrombin, as well as fibrinogen into fibrin, both of which are critical components in the formation of a blood clot [14].

The importance of calcium in human health continues to be evident in the functioning of the nervous system. Calcium plays an integral role in releasing neurotransmitters and propagation of action potential along the neurons, which is key for the overall functioning of the nervous system. This, in turn, affects all the processes that the nervous system controls, including sensations, movement, and even cognitive functions [15,16,17].

In addition to the roles mentioned earlier, calcium is involved in various other essential bodily functions. It helps maintain normal blood pressure, supports weight management, and plays a role in cellular functions, such as cell division and growth, influencing various hormones [18,19,20,21].

Calcium is a necessary nutrient that can be obtained from various dietary sources. It is commonly recognized that dairy items like milk, cheese, and yogurt are plentiful in calcium content. Other good sources include leafy green vegetables, such as kale and spinach, as well as certain fish, like salmon and sardines, especially when consumed with their edible bones. Additionally, many foods and drinks, such as orange juice, breakfast cereals, and plant-based milk alternatives, are often fortified with calcium to boost their nutritional value [22].

While these traditional sources are well-known, there is an often overlooked yet valuable source of dietary calcium—natural mineral waters. Mineral waters originate from geologically and physically protected underground water sources, and their mineral content, including calcium, remains constant. Natural calcium-rich mineral waters can be an excellent supplemental source of dietary calcium, especially for individuals with dairy allergies or intolerances, or those following plant-based diets [23].

Several factors make mineral waters a beneficial source of dietary calcium. First, the calcium in mineral water is highly bioavailable, and the body can easily absorb it. This is because mineral water typically has a balanced composition of minerals, creating a favorable environment for calcium absorption [24].

Second, natural calcium-rich mineral waters are free from calories, added sugars, and lactose, making them suitable for various dietary needs. They can be a precious source of calcium for individuals who are lactose intolerant and may struggle to meet their calcium needs through diet alone. Lastly, mineral waters are a practical and refreshing way to increase calcium intake. They can be easily integrated into daily habits, either consumed independently or used as a base for beverages such as tea or smoothies [25].

**Figure 1:**
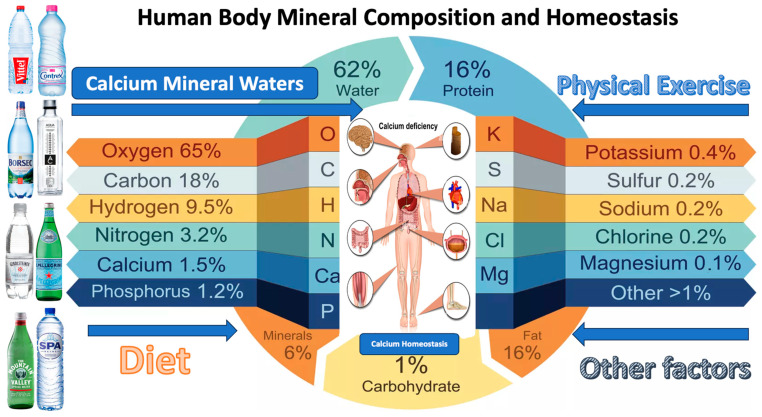
Calcium-rich mineral waters and reflected calcium health effects. The main calcium-rich mineral water sources existent on the market are presented in order to emphasize the variety of mineral compositions, identified by direct search, unbiased of commercial marketing or publicity.

This systematic review is dedicated to aggregating and examining the existing scientific research on the health and wellness benefits derived from natural calcium-rich mineral waters. It primarily focuses on calcium’s vital role in human health, the effectiveness of mineral water as a calcium source, and its implications on various health dimensions, including bone health, cardiovascular wellness, and weight management. This investigation also delves into the comparative bioavailability of calcium from mineral water and other dietary sources, understanding how efficiently our bodies can absorb and utilize this essential mineral (Figure 1). By analyzing global consumption trends and the distribution, this review underlines the potential worldwide impact of natural calcium-rich mineral waters. All these explorations aim to provide valuable insights to healthcare professionals, researchers, policymakers, and the public, paving the way for informed dietary guidelines, health policies, and future research directions.

## 2. Methods

**Figure 2:**
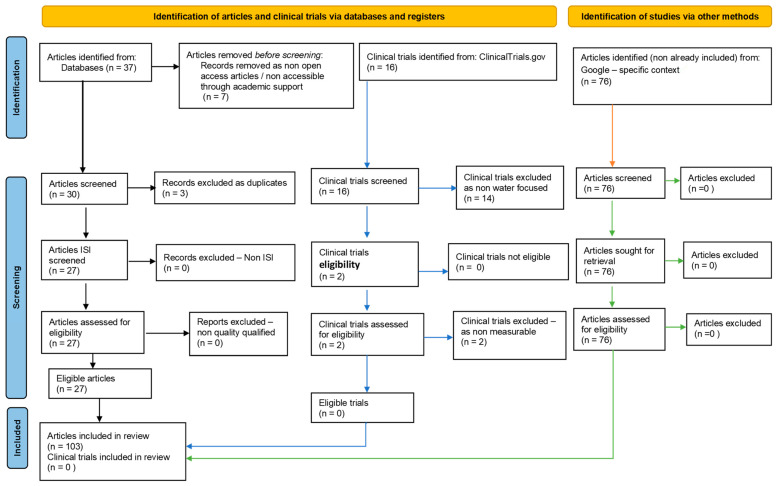
Adapted PRISMA flow diagram, customized for our study.

Our systematic review, performed under PRISMA guidelines, was recorded on PROSPERO ID: 439942. For this review, databases such as PubMed, Cochrane Library, Embase, Web of Science, and contextually, Google search may be selected as they are comprehensive and widely used in health and wellness research. The search strategy includes relevant keywords and medical subject headings (MeSH) related to calcium-rich mineral waters, their sources, and their impact on health and wellness: “mineral water,” “calcium,” “health,” “wellness,” “dietary calcium,” “natural mineral water”. Boolean operators like AND and OR have been used to combine or exclude keywords in the search strings. Our 103 references (27 from Databases search–Table 1) were obtained based on the above-described search strategy, after removing non-eligibles and duplicates. Separately, https://clinicaltrials.gov/ (accessed on 1 July 2023) was searched, obtaining 16 results, but no studies could be selected (Figure 2).

The inclusion criteria were as follows: studies examining natural calcium-rich mineral water consumption and its effects on health and wellness, published in English and peer-reviewed journals. Studies from all years were included to provide a comprehensive overview. Exclusion criteria included the following: non-English studies, studies not peer-reviewed (like editorials and opinion pieces), studies not focusing on natural calcium-rich mineral water, and studies not related to health or wellness effects.

## 3. Natural Calcium-Rich Mineral Waters Worldwide

Globally, numerous regions boast natural calcium-rich mineral waters, their unique mineral composition reflecting their sources’ specific geological and hydrological conditions. While these waters share the common feature of high calcium content, their other mineral constituents and sensory properties can vary widely, rendering each source distinctive [23]. Each of these waters offers unique health benefits with their distinct mineral compositions. The calcium in these waters is highly bioavailable, and other minerals can contribute to overall health and well-being [26,27].

Europe is particularly abundant in natural mineral waters [28]. In France, sources like Vittel and Contrex are notable for their high calcium and magnesium content. Italy has several calcium-rich mineral waters, including San Pellegrino and Uliveto. Germany also contributes to the list with Gerolsteiner, a naturally carbonated mineral water with a robust balance of calcium, magnesium, and bicarbonate [29].

In Romania, mineral waters have been used for their curative properties since ancient times. Towns such as Borsec and Sovata are famous for their healing waters and have been health tourism destinations for centuries. The consumption of these waters is deeply embedded in the local culture and traditional health practices [30]. Romania features calcium-abundant mineral waters, including ancient Borsec and springs in the Carpathian Mountains, such as AQUA Carpatica [31].

Across the Atlantic, North America hosts fewer calcium-rich mineral waters, mainly due to different geological formations. In the United States, the cultural significance of mineral water varies. In regions like the South and Midwest, mineral waters have historically been associated with health spas and resorts, like those in Hot Springs, Arkansas.

Today, while the consumption of mineral water is less culturally ingrained compared to Europe, mineral water is becoming increasingly popular as a healthy, natural beverage choice. However, sources such as the Mountain Valley Spring Water in Arkansas stand out for their balanced mineral profile, including calcium [32,33].

In Asia, countries like Japan, with their geothermal activity, house mineral-rich hot springs or “Onsen”, many of which have significant calcium content, which have a profound cultural and historical significance. Rich in minerals, these springs are cherished for their perceived healing properties and their role in relaxation and socialization [34]. India has a historical tradition of using mineral waters for therapeutic purposes. Certain mineral water sources, or ‘Tirtham’, are considered sacred in Hindu culture and used for religious rituals and purification. Over time, the consumption of bottled mineral water has risen due to health and safety concerns with local water supplies [35].

The consumption of mineral water has cultural and historical significance in many regions worldwide, reflecting local traditions, health beliefs, and the area’s geology. Europe has a long history of mineral water consumption, dating back to the Roman Empire when thermal and mineral springs were places of social gathering and regarded for their therapeutic properties. In countries such as Germany, France, and Italy, mineral water is not just a beverage but a lifestyle choice associated with wellness and good health. Spa towns, like Spa in Belgium or Baden-Baden in Germany, gained fame for their therapeutic waters, attracting visitors seeking wellness across Europe [36].

The mineral profile of these waters, largely influenced by the local geology, determines their specific characteristics. Calcium content varies substantially, generally within the range of 20 to 500 milligrams per liter (mg/L) but occasionally surpassing 1000 mg/L in particular sources [37]. This calcium is often in a bioavailable form that the human body can readily absorb, facilitated by the balance of other minerals and bicarbonate ions in the water [25,38]. The mineral content can also influence the water’s taste. While some maintain a neutral taste, others might adopt a slightly bitter or salty undertone due to minerals like calcium and magnesium. However, these flavors are typically subtle, sometimes enhancing the perceived freshness and palatability of the water [26,39].

It is worth mentioning that some regions of the world have waters with lower mineral content, including calcium. These regions include parts of the Pacific Northwest in the United States and areas with soft water, such as Scotland, Wales, and western England in the UK, Brazil’s Amazon Basin [40], and Southeast Asia [41,42].

Natural calcium-rich mineral waters worldwide offer a variety of mineral compositions, each unique to their source. Here are a few examples:

- Vittel (France, https://www.vittel.com/water-life (accessed on 20 June 2023)): Originating from the Vosges region of France, Vittel is known for its high calcium content. It contains approximately 240 milligrams of calcium per liter (mg/L). Alongside calcium, it also contains magnesium (about 42 mg/L) and bicarbonates.
- Contrex (France, https://www.fonsapor.com/products/contrex-500ml-natural-mineral-water-pet (accessed on 20 June 2023)): Another renowned mineral water from the French region of Vosges, Contrex stands out with one of the highest calcium contents among mineral waters, boasting about 468 mg/L. It also contains significant magnesium levels and is low in sodium.
- Gerolsteiner (Germany, https://www.gerolsteiner.de/fileadmin/Contentbilder/UnsereProdukte/Mineralwasser/Dokumente/gerolsteiner-sparkling-mineral-water-analysis.pdf (accessed on 20 June 2023)): Originating from the Volcanic Eifel region in Germany, Gerolsteiner mineral water offers a balanced mineral profile. It contains about 348 mg/L of calcium, 108 mg/L of magnesium, and a high bicarbonate content.
- AQUA Carpatica (Romania, https://aquacarpatica.com/products/ (accessed on 20 June 2023)): Sourced from the springs of the Carpathian Mountains, AQUA Carpatica contains about 260 mg/L of calcium. It also has a balanced magnesium level [31,43].
- Borsec (Romania, https://romaqua-group.ro/en/brands/borsec/ (accessed on 20 June 2023)): Known since ancient times, Borsec mineral water has approximately 195 mg/L of calcium. It is also characterized by a high content of bicarbonates [31,43].
- San Pellegrino (Italy, https://www.sanpellegrino.com/files/usa/2021_SP_WAR_EN.pdf (accessed on 20 June 2023)): San Pellegrino, sourced from the Italian Alps, is famous worldwide. It contains around 160 mg/L of calcium and is rich in bicarbonate and sulphate ions.
- Mountain Valley Spring Water (USA, https://www.mountainvalleyspring.com/pages/our-water (accessed on 20 June 2023)): Originating from the Ouachita Mountains in Arkansas, this water is balanced in mineral content, offering around 67 mg/L of calcium. It also contains other minerals like magnesium and potassium [44].
- Spa Reine (Belgium, https://mineralwaterfit.com/spa-reine-mineral-water-p-1828/ (accessed on 20 June 2023)): Spa Reine mineral water comes from the Ardennes in Belgium and has a lower, but still notable, calcium content of about 33 mg/L. Its unique mineral composition includes very low sodium levels, making it suitable for low-sodium diets [45].

Market trends and consumer attitudes towards calcium-rich mineral waters rise as consumer awareness about the importance of health and wellness grows; the natural calcium-rich mineral waters market has also risen substantially. Here are some of the key trends and attitudes shaping this market:

- Health-Conscious Consumers: As the importance of dietary calcium becomes more widely known, health-conscious consumers are actively seeking out beverages that can contribute to their daily mineral intake. Calcium-rich mineral waters fit this trend perfectly, offering a natural, calorie-free source of this vital mineral. The clear labelling of calcium content on packaging often influences purchasing decisions for these consumers [46].
- Natural and Organic Trend: Consumers today are increasingly seeking natural and organic products, driven by the perception that these are healthier and more environmentally friendly. Natural mineral waters, sourced directly from springs and untouched by human processing, are well-aligned with this trend. This preference for natural products has contributed to the growing popularity of natural mineral waters over other processed beverages [47].
- Sustainability and Eco-Friendliness: Sustainability is another significant trend in consumer attitudes. Consumers are becoming more aware of the environmental impact of their choices and are looking for products with sustainable packaging and sourcing. Mineral water companies that prioritize sustainable practices, from water sourcing to packaging, are more likely to gain favor with these eco-conscious consumers [48,49].
- Fitness and Hydration: With the growing popularity of fitness and outdoor activities, there is an increasing demand for hydrating beverages [50]. Mineral water, particularly calcium-rich mineral water, is perceived as a healthy hydrating choice providing essential minerals lost during physical exertion [51,52].

Regarding regional trends, Europe continues to be the leading consumer of bottled mineral water due to historical and cultural preferences. However, Asia-Pacific is the fastest-growing market due to rising health consciousness and increasing disposable income [53].

## 4. Dietary Calcium from Natural Mineral Waters

The bioavailability of calcium from mineral waters refers to the proportion of calcium absorbed by the body and used for physiological functions. Calcium bioavailability varies considerably between different dietary sources, and is affected by numerous factors, including the chemical form of calcium, other components in the food, and individual physiological factors. Numerous studies have investigated this topic, primarily focusing on comparing the bioavailability of calcium from mineral water to that of dairy products, traditionally recognized as a primary source of dietary calcium [54].

Several studies have suggested that the calcium in mineral water is at least as bioavailable as that dairy sources [55,56]. Factors contributing to the high bioavailability of calcium from mineral water may include the physical state of the calcium, the presence of other minerals, and the absence of inhibiting factors. Calcium in mineral water is dissolved, which may facilitate absorption in the gastrointestinal tract. The presence of bicarbonate in many mineral waters might also aid calcium absorption. Additionally, while some components like oxalates and phytates, which are naturally occurring in some plant foods, can inhibit calcium absorption, these are not present in mineral water. Oxalates are found in foods such as spinach, rhubarb, and certain types of beans. They bind with calcium in the digestive tract, preventing it from being absorbed into the body. Phytates, on the other hand, are found in whole grains, seeds, legumes, and some nuts. Similar to oxalates, they can bind with calcium and other minerals, reducing their bioavailability [57].

Some studies suggest that consuming calcium-rich mineral water can contribute significantly to overall calcium intake. For instance, one study estimated that drinking one liter of high-calcium mineral water could provide up to half the daily recommended calcium intake for adults [58].

It is important to note that the bioavailability of calcium can vary depending on individual factors such as age, physiological status, and overall diet. However, the evidence generally supports the conclusion that calcium from mineral water is highly bioavailable and can contribute meaningfully to daily calcium intake [59,60]. Calcium-rich mineral waters can have significant implications for individuals with dietary restrictions or specific health conditions, offering a viable and beneficial alternative source of calcium.

Lactose Intolerance and Dairy Allergies: People who are lactose intolerant or allergic to dairy products often struggle to meet their daily calcium needs, as dairy is one of the most common and bioavailable sources of dietary calcium. Calcium-rich mineral water can provide an excellent alternative for these individuals, as it is a lactose-free, hypoallergenic source of highly bioavailable calcium [61].

Vegetarian and Vegan Diets: Those following a vegetarian or, especially, a vegan diet can face challenges in achieving adequate calcium intake since plant-based calcium sources often have lower bioavailability due to inhibitors like oxalate and phytate [62]. Mineral calcium-rich waters offer an entirely plant-free source of this essential nutrient [63,64].

Osteoporosis and Bone Health: Adequate calcium intake is crucial for those with osteoporosis or other conditions affecting bone health. Since calcium-rich mineral water has a high bioavailability, it could be a useful addition to the diet for these individuals [65,66].

Kidney Stone Patients: Although calcium-rich foods are often discouraged for kidney stone patients, studies suggest that dietary calcium can help decrease the risk of stone formation, whereas calcium supplements may increase the risk. As a natural source of dietary calcium, mineral water could benefit these patients. However, this would depend on the specific composition of the water, as high levels of other minerals could influence stone risk [67].

People on Low-Sodium Diets: Many calcium-rich mineral waters have low sodium content, making them a suitable choice for individuals needing to limit their sodium intake [68].

It is important to note that while calcium-rich mineral water can be an excellent dietary addition, it should not replace a balanced diet or prescribed supplements for those with severe deficiencies or specific health conditions. As with any dietary changes, individuals with health concerns should discuss these with their healthcare provider. However, given the growing body of research supporting the high bioavailability of calcium in mineral water, it could be a valuable dietary component for many people [69].

## 5. Impact on Bone Health

Calcium intake heavily influences bone health, and research has been conducted to evaluate the effects of consuming calcium-rich mineral water on bone health [70]. Studies have examined the direct impact of calcium-rich mineral water consumption on bone metabolism markers [71]. For example, some research has shown that consuming mineral water high in calcium can reduce bone resorption markers, suggesting a potential protective effect against bone loss [72,73].

A study investigated the impact of mineral water consumption on bone health in postmenopausal women, a group particularly at risk for osteoporosis due to decreased estrogen levels, and found that those who consumed mineral water containing high levels of calcium had improved markers of bone metabolism compared to those who did not [74].

Research has also explored the impact of consuming calcium-rich mineral water on bone density. A study conducted on rats demonstrated that those receiving mineral water high in calcium had a higher bone mineral density compared to those who did not receive this mineral water. While human studies are more complex, this does suggest potential benefits for bone health [75]. Additionally, some studies have considered the benefits of calcium-rich mineral water for individuals who have difficulty consuming traditional calcium sources. For instance, lactose-intolerant individuals or those on vegan diets who drank calcium-rich mineral water were found to have improved calcium levels, suggesting an overall positive effect on bone health [76]. The mechanisms through which calcium-rich mineral water influences health, particularly bone health, can be viewed from two main perspectives: the bioavailability of calcium in mineral water and its subsequent physiological effects.

Bioavailability: As previously discussed, the calcium in mineral water is highly bioavailable. When you consume mineral water, the calcium it contains is in a dissolved ionic form, readily absorbed in the intestine. This easy absorption could increase the overall calcium available to the body [41,56].

Absorption Enhancement: Some mineral waters contain additional minerals like magnesium and bicarbonate. There is evidence to suggest that these can enhance calcium absorption. For instance, bicarbonate may reduce the acidity in the stomach, which in turn could improve calcium absorption [65].

Bone Mineralization: Once calcium is absorbed into the bloodstream, it can be used for various physiological functions. A significant amount of this calcium is utilized for bone mineralization, a process that maintains the strength and structure of bones. When the body has an adequate supply of calcium, this process can proceed optimally, leading to healthier bones [70].

Inhibition of Bone Resorption: Evidence suggests that calcium-rich mineral water could inhibit bone resorption, the process by which bones are broken down, and the minerals within them, including calcium, are released into the bloodstream. Providing a ready supply of calcium, mineral water could help reduce the need for this process, thereby potentially reducing bone loss [66].

Balancing Calcium Levels: The body finely regulates calcium levels. When blood calcium levels are low, the body can respond by increasing calcium absorption in the gut, reducing calcium excretion in the kidneys, and releasing calcium from the bones. By contributing to overall calcium intake, calcium-rich mineral water could help maintain balanced calcium levels, reducing the need for the body to draw on its own calcium stores [77]. However, the exact effects could depend on various factors, including individual physiological differences and the specific composition of the mineral water. Further research is required to fully understand these mechanisms and their implications for health and wellness.

## 6. Cardiovascular Implications

The relationship between calcium intake, particularly from supplements, and cardiovascular health has been a considerable research and debate topic. While some studies suggest that high calcium intake could lead to cardiovascular issues like heart disease or stroke, others suggest no such relationship or potential benefits [78,79,80].

In the context of calcium-rich mineral water, the body of research is less extensive, but some studies have explored this relationship. It is important to note that the findings from these studies should be interpreted cautiously due to factors such as limited sample sizes, different methodologies, and variability in the mineral compositions of the waters studied. The mechanisms by which calcium-rich mineral water might influence cardiovascular health are multifaceted and interconnected, involving direct and indirect effects [81,82].

Blood Pressure Regulation: Some studies have suggested that calcium can play a role in regulating blood pressure. Studies have shown that individuals who consumed mineral water with a high calcium content experienced a slight decrease in blood pressure. However, the results are inconsistent across all studies, and more research is needed to confirm these findings. Calcium is crucial in muscle contraction and relaxation, including the muscles lining the blood vessels. Increased dietary calcium can lead to the dilation of these vessels, potentially resulting in lowered blood pressure. Furthermore, calcium is also involved in transmitting nerve signals, which can influence heart rate and blood pressure [83].

Heart Disease Risk Factors: Some evidence suggests that calcium-rich mineral water could influence certain risk factors for heart disease. For example, one study found that the consumption of mineral water high in calcium was associated with reduced LDL (“bad”) cholesterol levels and increased HDL (“good”) cholesterol levels. However, the precise mechanisms behind these observations remain unclear [23,84,85].

Vascular Calcification: One concern raised about high calcium intake, particularly from supplements, is that it could contribute to vascular calcification, a process that can lead to heart disease. However, studies suggest that calcium from dietary sources, including mineral water, does not have the same effect. Some researchers hypothesize that the calcium in food and water is absorbed more gradually, reducing the risk of calcification [86,87,88].

Inhibition of Vascular Calcification: While excessive calcium intake from supplements has been associated with an increased risk of vascular calcification, calcium from dietary sources appears to have a different effect. This could be due to the slower and more regulated absorption of dietary calcium, which might prevent sudden increases in blood calcium levels that can promote calcification [89].

Electrolyte Balance: Calcium is a key player in maintaining the balance of electrolytes in the body. Electrolyte imbalances can negatively affect heart rhythm. Regular consumption of calcium-rich mineral water could contribute to keeping this balance. Calcium is a vital electrolyte and helps maintain the overall balance of electrolytes in the body. Electrolytes are critical for many bodily functions, including maintaining the balance of fluids, transmitting nerve signals, and regulating muscle function, including the heartbeat [90].

Cholesterol Metabolism: Calcium binds to fatty acids and bile in the gut, influencing fat digestion and absorption. This can result in a decrease in the level of low-density lipoprotein (LDL, or “bad”) cholesterol and an increase in high-density lipoprotein (HDL, or “good”) cholesterol levels in the blood [91].

Mineral waters often contain other minerals in addition to calcium, such as magnesium and potassium, which are known to have beneficial effects on cardiovascular health. The presence of these minerals could contribute to the potential cardiovascular benefits of consuming mineral water. While there are some indications that drinking calcium-rich mineral water could have benefits for cardiovascular health, the evidence is not yet strong or consistent enough to draw definitive conclusions. Further, well-controlled studies are needed to explore this potential relationship in more detail. As always, it is important to consider overall dietary patterns and lifestyle factors, which play a significant role in cardiovascular health. While these mechanisms could help explain the potential cardiovascular benefits of consuming calcium-rich mineral water, it is important to note that the current body of research is limited, and these mechanisms are based on a broader understanding of calcium’s role in the body. More specific research on calcium from mineral water and cardiovascular health is needed to confirm these mechanisms and fully understand their potential benefits [23].

## 7. Role in Weight Management

While the connection between calcium-rich mineral water and weight management is not as extensively studied as its relationship with bone or cardiovascular health, a few studies hint at potential benefits. In some observational studies, higher calcium intake, including from water sources, has been associated with lower body weight and less weight gain over time. For example, one study suggested that individuals who consumed more calcium-rich mineral water had a lower risk of developing overweight and obesity [82,92].

Some research suggests that calcium might help regulate appetite, potentially influencing weight management. A study found that participants who consumed mineral water high in calcium reported feeling more satiated and had reduced hunger ratings compared to those who did not consume this water [93,94].

Calcium plays a role in the body’s metabolism of fat. It is suggested that higher calcium intake could increase fat excretion, which means the body absorbs less fat. Additionally, calcium might help break down body fat, thus aiding weight loss. There is some evidence that calcium could help regulate appetite, appetite suppression, potentially through effects on hormones that control hunger and satiety. This could lead to reduced calorie intake and contribute to weight management [95].

Thermogenesis Stimulation: Some studies suggest that calcium might stimulate thermogenesis, the body’s heat production process. This could potentially increase energy expenditure and support weight management [96,97].

While there are plausible mechanisms through which calcium could influence weight, it is important to note that weight management is influenced by a wide array of factors, including total calorie intake, physical activity, genetics, and other lifestyle and environmental factors [98]. More research is needed to definitively establish the role of calcium-rich mineral water in weight management. A balanced diet and regular physical activity are the cornerstones of healthy weight management.

## 8. Other Potential Health Benefits

While the primary focus of research on calcium-rich mineral waters has been on bone health, cardiovascular health, and weight management, a few additional health benefits associated with their consumption have been identified in the literature.

Calcium-rich mineral water may provide relief for individuals with certain digestive disorders. For example, high-calcium mineral waters may have antacid properties [99] and help neutralize stomach acid, relieving individuals with acid reflux or heartburn. For example, some studies have found that it may help alleviate symptoms of constipation by promoting bowel movement and improving overall gut health [55,100].

Hydration: Staying adequately hydrated is crucial for overall health, and mineral waters can contribute to hydration while providing essential minerals [84]. The minerals present in calcium-rich mineral water, such as calcium and magnesium, can aid in maintaining electrolyte balance, which is essential for optimal hydration [4,23,55,65,100].

Adequate mineral intake is important for optimal exercise performance. Calcium, along with other minerals, plays a vital role in muscle contraction and relaxation. Some studies suggest that calcium-rich mineral water, due to its bioavailability and mineral content, may aid in muscle function and enhance exercise performance [56,66].

Kidney Stone Prevention: Despite concerns about calcium intake and kidney stones, some studies suggest that consuming calcium-rich mineral water may help reduce the risk of certain types of kidney stones [101]. This is because the additional minerals in the water, along with increased hydration, may promote urinary dilution and reduce the concentration of stone-forming substances [102,103].

It is important to note that while these potential health benefits have been reported, the evidence is still limited, and more research is needed to fully understand the extent and mechanisms behind these effects. While this review extensively examines the health and wellness benefits of calcium-rich mineral waters, it is crucial to acknowledge the potential side effects of high calcium intake as well. Consuming too much calcium, especially in the form of supplements or excessively mineral-rich water, can lead to hypercalcemia. This condition is characterized by elevated levels of calcium in the blood, which may cause kidney stones, renal insufficiency, or impaired absorption of other minerals such as iron and zinc. Additionally, excessive calcium intake could lead to constipation and might also interfere with the absorption of other nutrients, such as iron, magnesium, and zinc. Consequently, while calcium-rich mineral waters can be beneficial for bone health and overall wellness, they should be consumed in moderation and within recommended daily intake levels to prevent potential health risks.

## 9. Conclusions

Calcium-rich mineral waters present promising avenues for enhancing health outcomes, though there is a critical need for additional research to thoroughly comprehend their effects and elaborate consumption guidelines. Highlighting the necessity for more comprehensive studies, the potential health influences of these mineral waters should be elucidated in future explorations. Previous studies primarily included average adult populations, leaving a gap for research focusing on particular groups such as children, the elderly, or individuals with specific health conditions like osteoporosis or heart disease. These studies would help establish if the possible benefits of mineral water consumption differ across diverse age brackets and health conditions. Upcoming research should dig deeper into the unique mineral compositions of these waters, employ long-term investigation strategies, include a broad spectrum of population groups, and leverage standardized methodologies.

## Figures and Tables

**Table 1:** Synthesis of eligible studies selected from databases.

No.	Article	Year	Main Details
1	Vannucci L, Fossi C, Quattrini S, Guasti L, Pampaloni B, Gronchi G et al. Calcium Intake in bone health: A focus on calcium-rich mineral waters. Nutrients	2018	The study investigates the crucial role of calcium in human bone health, particularly in bone mineralization. It further delves into the significance of calcium-rich mineral waters, focusing on the high bioavailability of calcium they provide.
2	Flynn A. The role of dietary calcium in bone health. Proc Nutr Soc	2003	Calcium, predominantly found in bones, is crucial for their development and maintenance. Its dietary intake varies across life stages, with disagreements on optimal amounts. Insufficiency can impair bone health and potentially lead to osteoporosis.
3	Tandoǧan B, Ulusu NN. Importance of calcium. Turkish J Med Sci.	2005	Calcium, the body’s most abundant mineral, regulates numerous processes, including muscle function, fibrin polymerisation, and nervous impulses. Managed by calcium-transporting systems, its balance is vital as fluctuations can prompt varied responses, including apoptosis.
4	Rusoff LL. Calcium–Osteoporosis and Blood Pressure. J Dairy Sci.	1987	Optimum calcium intake throughout life, primarily through dairy, is vital for peak bone mass development and preventing osteoporosis. Unfortunately, many, particularly older females, fall short of the recommended intake, risking bone diseases and high blood pressure.
5	Shkembi B, Huppertz T. Calcium absorption from food products: Food matrix effects. Nutrients.	2022	Calcium absorption from food varies significantly due to interactions with other food components and factors like fermentation and gastrointestinal conditions. Absorption requires calcium to be ionized, facilitated by the stomach’s low pH.
6	Quattrini S, Pampaloni B, Brandi ML. Natural mineral waters: Chemical characteristics and health effects. Clin Cases Miner Bone Metab.	2016	Drinking 1.5–2 L of water daily is vital for maintaining body water equilibrium. Despite concerns over plastic containers, natural mineral waters’ unique mineral compositions can offer numerous health benefits, impacting various physiological and pathological conditions.
7	Albertini MC, Dachà M, Teodori L, Conti ME. Drinking mineral waters: Biochemical effects and health implica-tions—The state-of-the-art. Int J Environ Heal.	2007	While the health benefits of mineral waters have been shown to have significant biochemical implications, adverse effects must also be considered. This review underscores the need for further research to ensure public health safety and avoid mineral water misuse.
8	Heaney RP. Absorbability and utility of calcium in mineral waters. Am J Clin Nutr.	2006	This research investigates the potential of high-calcium mineral waters to address calcium deficiency in North America. The absorbability of calcium from high-mineral waters was measured in human volunteers and compared to calcium from milk.
9	Bacciottini L, Tanini A, Falchetti A, Masi L, Franceschelli F, Pampaloni B et al. Calcium bioavailability from a calcium-rich mineral water, with some observations on method. J Clin Gastroenterol.	2004	This study compares the bioavailability of calcium from high-calcium mineral water and milk in 27 healthy subjects. Results indicate that the calcium from the mineral water is highly bioavailable, comparable to milk calcium. The research underscores mineral water as a potential significant source of dietary calcium.
10	Bourassa MW, Abrams SA, Belizán JM, Boy E, Cormick G, Quijano CD et al. Interventions to improve calcium intake through foods in populations with low intake. Ann N Y Acad Sci.	2022	In countries with low calcium intake, food-based solutions can enhance calcium consumption and bioavailability. Strategies include promoting calcium-rich animal-source foods and plant foods, improving calcium content via food processing techniques, fortifying staple foods with calcium, and exploring biofortification.
11	Weaver CM, Proulx WR, Heaney R. Choices for achieving adequate dietary calcium with a vegetarian diet. Am J Clin Nutr.	1999	While dairy is common in America, certain plants also provide calcium. Yet, a purely plant-based diet may need fortified foods or supplements for sufficient calcium.
12	Pampaloni B, Brandi ML. Mineral water as food for bone: an overview. Int J Bone Fragility.	2022	Natural mineral waters, particularly calcium-rich ones (>150 mg/L), provide bioavailable calcium and other micronutrients beneficial for bone health. Despite the potential negative effects from plasticizers in bottled waters, their consumption is common.
13	Burckhardt P. The effect of the alkali load of mineral water on bone metabolism: Interventional studies. J Nutr.	2008	Alkali supplements and diets reduce bone resorption and increase bone mineral density. Bicarbonate-rich alkali mineral waters with low acid load effectively lower bone resorption markers and parathyroid hormone levels, surpassing acidic calcium-rich mineral waters, regardless of sufficient calcium intake.
14	Guillemant J, Le HT, Accarie C, Du Montcel ST, Delabroise AM, Arnaud MJ et al. Mineral water as a source of dietary calcium: Acute effects on parathyroid function and bone resorption in young men. Am J Clin Nutr.	2000	The study aimed to investigate the effectiveness of high-calcium mineral water as an additional source of dietary calcium. Results showed that intake of high-calcium water significantly reduced parathyroid hormone secretion and bone resorption markers, indicating its potential in inhibiting bone resorption.
15	Wynn E, Krieg MA, Aeschlimann JM, Burckhardt P. Alkaline mineral water lowers bone resorption even in calcium sufficiency: Alkaline mineral water and bone metabolism. Bone.	2009	In this study, the effects of an alkaline mineral water rich in bicarbonate and an acid mineral water rich in calcium were compared in young women with normal calcium intake. The alkaline water led to a significant decrease in parathyroid hormone and bone resorption markers, while the acid water had no effect on bone resorption.
16	Meunier PJ, Jenvrin C, Munoz F, De La Gueronnière V, Garnero P, Menz M. Consumption of a high calcium mineral water lowers biochemical indices of bone remodeling in postmenopausal women with low calcium intake. Osteoporos Int.	2005	In a 6-month trial, postmenopausal women with low calcium intake were given a high-calcium mineral water (HCaMW) or a low-calcium placebo water. The HCaMW group experienced significant decreases in serum parathyroid hormone and biochemical markers of bone remodeling, indicating the potential benefits in repairing calcium deficiency and reducing age-related bone loss.
17	Y, Xu A, Qiu Z, Wang L, Wang J, Luo J et al. Drinking Natural Mineral Water Maintains Bone Health in Young Rats With Metabolic Acidosis. Front Nutr.	2022	The rats drinking bicarbonate-rich natural mineral water showed improved bone health, including higher bone mineral density, greater bone microstructure, and increased bone strength. Drinking natural mineral water, especially bicarbonate-rich water, can effectively improve bone health in individuals with metabolic acidosis.
18	Wang L, Manson JAE, Sesso HD. Calcium intake and risk of cardiovascular disease: A review of prospective studies and randomized clinical trials. Am J Cardiovasc Drugs.	2012	Adequate calcium intake is crucial for bone health and has potential effects on cardiovascular disease (CVD). Experimental studies suggest calcium’s involvement in cardiovascular processes. Epidemiological studies show mixed results
19	Myung SK, Kim HB, Lee YJ, Choi YJ, Oh SW. Calcium supplements and risk of cardiovascular disease: A me-ta-analysis of clinical trials. Nutrients.	2021	This meta-analysis of double-blind, placebo-controlled trials found that calcium supplements increased the risk of CVD and coronary heart disease (CHD) by approximately 15% in healthy postmenopausal women. Subgroup analysis showed that both dietary and supplementary calcium intake were associated with increased CVD and CHD risk.
20	Tankeu AT, Ndip Agbor V, Noubiap JJ. Calcium supplementation and cardiovascular risk: A rising concern. J Clin Hypertens.	2017	The use of calcium supplementation has increased globally, driven by its established role in osteoporosis prevention and treatment. However, emerging evidence suggests potential adverse cardiovascular effects.
21	Böhmer H, Müller H, Resch KL. Calcium supplementation with calcium-rich mineral waters: A systematic review and meta-analysis of its bioavailability. Osteoporos Int.	2000	The relevance of calcium in preventing and treating osteoporosis is well established. Higher daily calcium intake is recommended, but achieving it can be challenging. Calcium-rich mineral waters may offer a promising alternative, as they have shown comparable or better calcium bioavailability than dairy products.
22	Nerbrand C, Agréus L, Lenner RA, Nyberg P, Svärdsudd K. The influence of calcium and magnesium in drinking water and diet on cardiovascular risk factors in individuals living in hard and soft water areas with differences in cardiovascular mortality. BMC Public Health.	2003	Current recommendations for daily calcium intake are being questioned, with new guidelines suggesting higher levels that may be challenging to meet through traditional sources like dairy products or supplements. Calcium-rich mineral waters could provide a promising alternative. A systematic review and meta-analysis found that calcium absorption from mineral waters was significantly higher than from dairy products.
23	Anderson JJB, Klemmer PJ. Risk of high dietary calcium for arterial calcification in older adults. Nutrients.	2013	As the kidneys have limited capacity to eliminate excess calcium, the risk of soft-tissue calcification may increase, especially in older adults with reduced renal function. While maintaining bone health remains important, policy recommendations for calcium intake in adults should also consider the potential risks of cardiovascular diseases associated with excessive calcium intake.
24	Phillips-Eakley AK, McKenney-Drake ML, Bahls M, Newcomer SC, Radcliffe JS, Wastney ME et al. Effect of High-Calcium Diet on Coronary Artery Disease in Ossabaw Miniature Swine With Metabolic Syndrome. J Am Heart Assoc.	2015	This study aimed to investigate the impact of high calcium intake on coronary artery calcification using innovative calcium tracer kinetic modeling in pigs with diet-induced metabolic syndrome. The results showed no detectable effect of high calcium diets on coronary artery calcium deposition. Secondary endpoints also demonstrated no treatment differences in coronary artery disease or function.
25	Anderson JJB, Kruszka B, Delaney JAC, He K, Burke GL, Alonso A et al. Calcium intake from diet and sup-plements and the risk of coronary artery calcification and its progression among older adults: 10-year follow-up of the multi-ethnic study of atherosclerosis (MESA). J Am Heart Assoc.	2016	In a longitudinal cohort study, the relationship between calcium intake (from both foods and supplements) and coronary artery calcification (CAC) was assessed. The study included 5448 adults without clinically diagnosed CVD. Results showed that high total calcium intake, obtained from dietary sources, was associated with a decreased risk of incident atherosclerosis over long-term follow-up.
26	Aptel I, Cance-Rouzaud A, Grandjean H. Association between calcium ingested from drinking water and femoral bone density in elderly women: Evidence from the EPIDOS cohort. J Bone Miner Res.	1999	In the EPIDOS multicenter study, data from 4434 women over 75 years old were analyzed to examine the relationship between dietary calcium, calcium from drinking water, and bone density at the femoral neck. Total calcium intake showed a significant correlation with bone density. Specifically, a 100 mg/day increase in calcium from drinking water was associated with a 0.5% increase in femoral bone density.
27	Teegarden D, Gunther CW. Can the controversial relationship between dietary calcium and body weight be mechanistically explained by alterations in appetite and food intake? Nutr Rev.	2008	Studies have suggested that calcium or dairy products may affect body weight and fat by influencing appetite and food intake. However, recent research has found no evidence of complete compensation for increased energy intake from dairy products, indicating that a short-term increase in dairy intake does not affect appetite. Additionally, altering the calcium content of a meal has shown no impact on appetite-related hormones or energy intake from subsequent meals.

## Data Availability

Not applicable.
